# Are Microalbuminuria and Elevated 24 H Urinary Microalbumin Excretion within Normal Range Associated with Metabolic Syndrome in Chinese Adults?

**DOI:** 10.1371/journal.pone.0138410

**Published:** 2015-09-17

**Authors:** Zeng Ge, Xiaolei Guo, Xiaorong Chen, Jiyu Zhang, Liuxia Yan, Junli Tang, Xiaoning Cai, Aiqiang Xu, Jixiang Ma

**Affiliations:** 1 National Center for Chronic and Noncommunicable Disease Control and Prevention, Chinese Center for Disease Control and Prevention, Beijing, China; 2 Shandong Center for Disease Control and Prevention, Jinan, China; The Ohio State University, UNITED STATES

## Abstract

The association of microalbuminuria and elevated 24 h urinary microalbumin excretion (UAE) with metabolic syndrome (MetS) has not been well examined in Chinese Adults. In the present study, a population-based cross-sectional study was conducted among Chinese adults aged 18–69 years in Shandong province in 2011 to determine the relationship between them. Data on 24 h UAE and other variables were obtained and components of MetS were examined. The prevalence of MetS and mean 24 h UAE was 24.7% and 6.7 mg, respectively. Compared with participants with normoalbuminuria, the odds of MetS and its components including central obesity, elevated blood pressure and elevated fasting glucose, but not the odds of low HDL cholesterol and elevated triglycerides, significantly increased in those with microalbuminuria. Additionally, the odds of MetS and its three components significantly increased across increasing quartiles of 24 h UAE within normal range (1.00, 1.11, 1.02 and 1.58, respectively, for MetS; 1.00, 1.14, 1.21, and 1.68, respectively, for central obesity; 1.00, 1.35, 1.26 and 1.68, respectively, for elevated blood pressure; and 1.00, 1.32, 1.06 and 1.47, respectively, for elevated triglycerides; p for linear trend ≤0.05 for all). Furthermore, for each increment of 10 mg 24 h UAE within normal range, the odds of MetS and its components including central obesity, elevated blood pressure, and elevated triglycerides significantly increased by 53%, 65%, 55%, and 41%, respectively. These findings suggest that both microalbuminuria and elevated 24 h UAE within normal range might be important risk factors for MetS in Chinese adults.

## Introduction

Metabolic syndrome (MetS) is a serious public health challenge in China and worldwide because of its high incidence and concomitant risks of cardiovascular disease and all-cause mortality[1, 2]. Epidemiological studies have been conducted globally to determine the relationship between microalbuminuria estimated by an early morning or random spot urine sample and risk of MetS, and a significant positive correlation was found between them[3–10]. However, detailed analyses evaluating the risk of 24 h urinary microalbumin excretion (UAE) on MetS have been limited[6]. In the meantime none of population-based studies has specifically examined the effect of 24 h UAE on the risk of MetS among Chinese adults.

Therefore, in this study, we analyzed the group data from a cross sectional study of Chinese adults to investigate the association of microalbuminuria and elevated 24 h UAE within normal range with the risk of MetS in Chinese adults.

## Methods

### Participants

In 2011, the Shandong and Ministry of Health Action on Salt and Hypertension (SMASH) project was conducted. Details of the study design and definition of important variables, including high school education, alcohol consumption, smoking, and leisure physical activities, have been described elsewhere[11]. Briefly, a four-stage stratified sampling method was used to select a provincially representative sample of the general adult population aged 18 to 69 years in China. 2112 participants were randomly selected and invited to provide 24-hour urine samples. Finally, 1904 participants were identified and retained for this analysis, after we excluded 88 participants with incomplete 24-hour urine collections, 2 with 24 h UAE >300 mg and 118 missing other variables of interest. The study was approved by the Shandong Center for Disease Control and Prevention Ethics Committee. All study participants gave written informed consent.

### Data collection

Information on variables, including demographic characteristics, smoking, alcohol consumption, leisure-time physical activity, as well as previous diagnosis and treatment of hypertension and diabetes, was collected at local health stations, in community clinics, or by home visits by specially trained research staff using a standard questionnaire.

Three blood pressure measurements were obtained in the sitting position after at least 5 min of rest by trained investigators. Body weight and height were measured with participants wearing light indoor clothing without shoes during clinical examination, and body mass index (BMI) was calculated as weight in kilograms divided by height in square meters. Waist circumference was measured at 1 cm above the navel at minimal respiration.

Overnight fasting blood specimens (≥10 h) were obtained at the examination centers and shipped to Jinan ADICON Clinical Laboratory where the measurements of fasting plasma glucose, triglycerides, as well as high density lipoprotein (HDL) cholesterol were performed. Plasma glucose was measured using a modified hexokinase enzymatic method. Serum cholesterol and triglyceride levels were analysed enzymatically using commercially available reagents.

Additionally, a single 24-hour urine sample was collected from each participant and also shipped to Jinan ADICON Clinical Laboratory to be analyzed for creatinine determined by using the picric acid method and 24 h UAE assessed by using an immunonephelometric method (coefficient of variation was 3.0%) with Olympus AU640 Analyzer. Microalbuminuria was defined as 24 h UAE between 30 and 300 mg[12]. The completeness of urine collections was validated by urine volume and urinary creatinine. Collections with urine volume ≥500ml and urinary creatinine within gender-specific mean ± 2×standard deviation (1.91 to 18.27 mmol for men; 1.36 to 14.28 mmol for women) were considered complete[11].

### Definition of MetS

MetS was defined according to the harmonized criteria as the presence of three or more of the following risk factors[13]: (1) central obesity: waist circumference ≥90 cm for Chinese men and ≥80 cm for Chinese women; (2) elevated blood pressure: systolic blood pressure ≥130 mmHg and/or diastolic blood pressure ≥85 mmHg, or antihypertensive drug treatment for patient with hypertension; (3) elevated triglycerides: fasting plasma triglycerides ≥1.7 mmol/L, or drug treatment for elevated triglycerides; (4) reduced HDL cholesterol: fasting HDL cholesterol <1.0 mmol/L in men and < 1.3 mmol/L in women, or drug treatment for reduced HDL cholesterol; and (5) elevated fasting glucose: fasting glucose level of ≥5.6 mmol/L, or drug treatment for elevated glucose.

### Statistical analysis

Data are expressed as either the mean (SD) for continuous variables or percentages for categorical variables. Study participants were classified into four categories according to their quartiles of 24 h UAE (<4.40, 4.40–5.89, 5.90–8.09 and ≥8.10 mg).

Logistic regression models were applied to estimate odds ratios (ORs) and 95% confidence intervals (CIs) for odds of MetS according to 24 h UAE within normal range, adjusted for age, sex, high school education, urbanization, leisure-physical activity, alcohol consumption, and smoking. Participants who were with normoalbuminuria or 24 h UAE<4.40 mg were used as reference groups for those analyses estimating ORs and 95% CIs. The presence of a linear trend was tested by using the medians of the average 24 h UAE in each group treated as a continuous variable in the logistic regression models or by treating 24 h UAE as a continuous variable in linear regression models.

All statistical analyses were conducted using SAS statistical software version 9.3 (SAS Institute Inc., Cary, North Carolina, USA). All tests were two-sided, and a P value of less than 0.05 was considered to indicate statistical significance.

## Results

The baseline characteristics of study participants according to microalbuminuria and quartiles of 24 h UAE within normal range are presented in Table 1. On average, of 1904 participants, 77 (4.0%) were with microalbuminuria. Participants with microalbuminuria were more likely to have higher mean systolic and diastolic blood pressure, waist circumference, BMI, and FBG compared those with normoalbuminuria. In general, mean diastolic blood pressure, waist circumference, BMI, and triglycerides increased with increasing quartiles of 24 h UAE within normal range.

**Table 1:** Baseline characteristics of participants according to microalbuminuria and quartiles of 24 h UAE within normal range.

	Mcroalbuminuria		p	24 h UAE (mg)				p
	No	Yes		Q1	Q2	Q3	Q4	
Number of participants	1827	77		458	441	475	453	
Men (%)	52.8	48.1	0.41	55	53.1	52	51.2	0.23
Age (years)	41.2(13.9)	44.2(14.1)	0.07	40.8(14.2)	40.4(14.1)	42.4(13.9)	41.1(13.5)	0.33
SBP (mmHg)	120.8(18.0)	131.1(23.2)	<0.001	119.5(17.0)	121.0(17.7)	120.7(18.2)	122.0(19.1)	0.06
DBP (mmHg)	78.5(11.2)	85.2(13.7)	<0.001	77.5(10.5)	78.8(10.8)	78.2(11.1)	79.4(12.3)	0.02
WC (cm)	24.4(3.8)	25.9(4.5)	0.002	82.2(11.5)	83.3(10.9)	83.4(10.8)	84.7(11.1)	<0.001
BMI (kg/cm^2^)	83.4(11.1)	87.6(12.0)	0.009	24.0(3.7)	24.4(4.0)	24.3(3.6)	25.0(3.9)	<0.001
High-school graduate (%)	25.18	22.08	0.54	24.2	24.7	26.5	25.2	0.61
Smoking (%)	31.36	31.17	0.97	37.6	32	27.6	28.5	0.001
Alcohol consumption (%)	41.76	33.77	0.16	36.2	37	37.5	37.8	0.62
Leisure-physical activity (%)	19.81	20.78	0.84	17.9	18.6	20.8	21.9	0.09
Urban (%)	32.62	31.17	0.79	25.8	31.5	34.5	38.6	<0.001
FBG (mmol/L)	5.5(1.1)	6.0(2.1)	0.02	5.5(1.2)	5.4(0.8)	5.5(1.1)	5.5(1.3)	0.42
TG (mmol/L)	1.4(1.6)	1.8(2.0)	0.10	1.3(1.2)	1.4(1.3)	1.4(1.9)	1.5(1.8)	0.027
HDL (mmol/L)	1.4(0.3)	1.4(0.4)	0.46	1.4(0.3)	1.4(0.3)	1.4(0.3)	1.4(0.4)	0.54
24 h UAE (mg)	6.9(4.0)	76.9(53.3)	<0.001	3.5(0.7)	5.1(0.4)	6.8(0.6)	12.3(4.5)	<0.001

Data were the mean (SD) for continuous variables, or percentages for categorical variables.

Abbreviation: 24 h UAE, 24 h urinary microalbumin excretion; SBP, systolic blood pressure; DBP, diastolic blood pressure; WC, waist circumference; BMI, body mass index; FBG, fasting blood glucose; TG, triglycerides; HDL, high-density lipoprotein cholesterol

**Fig 1:**
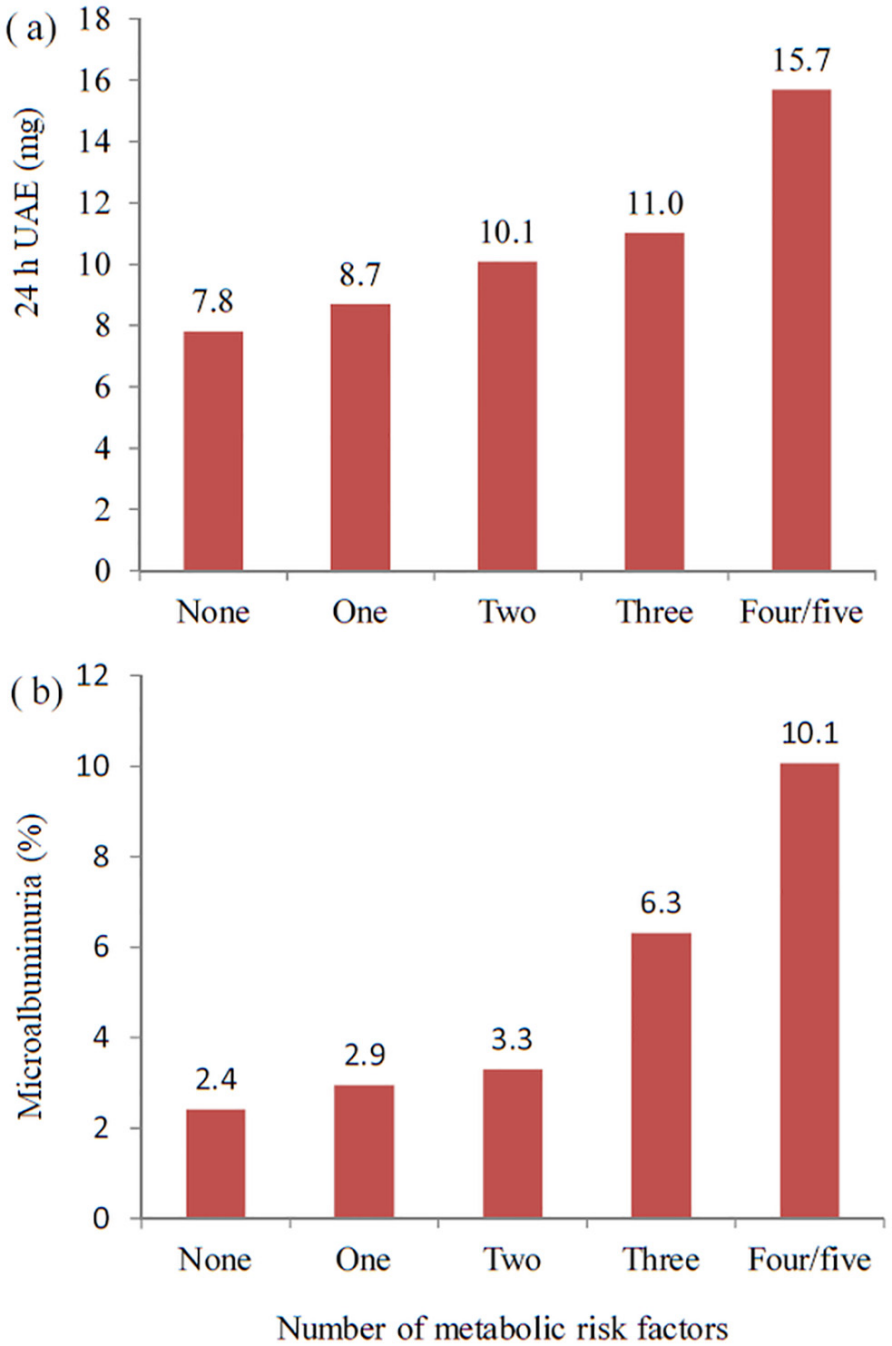
Mean 24 h UAE (a) and prevalence of microalbuminuria (b), according to number of metabolic risk factors.

For all participants, the mean 24 h UAE was 6.7 mg. The mean 24 h UAE for participants with none (n = 498), one (n = 511), two (n = 426), three (n = 302), and four or five (n = 169) of metabolic risk factors was 7.8, 8.7, 10.1, 11.0, and 15.7 mg, respectively. The corresponding prevalence of microalbuminuria was 2.4, 2.9, 3.3, 6.3, and 10.1%. In aggregate, both mean 24 h UAE and prevalence of microalbuminuria were significantly increased with the number of metabolic risk factors (*P* values for linear trend <0.001 for both, Fig 1).

Age and multivariate-adjusted ORs (95%CIs) of MetS and its components by microalbuminuria are shown in Table 2. As expected, microalbuminuria was a strong independent predictor of the risk of MetS. Compared with participants with normoalbuminuria, the age and multivariate-adjusted ORs (95%CIs) for MetS were 2.68(1.67, 4.29) and 2.71(1.69, 4.36) for those with microalbuminuria. As for components of MetS, odds of central obesity, elevated blood pressure, and elevated triglycerides were significantly higher among participants with microalbuminuria than those with normoalbuminuria.

**Table 2:** Adjusted OR of metabolic syndrome and its components by microalbuminuria (Odds ratios and 95% confidence intervals).

	No. of cases	Normoalbuminuria	Microalbuminuria	
			Model 1†	Model 2‡
Metabolic syndrome	470	1.00	2.68(1.67, 4.29)	2.71(1.69, 4.36)
Central obesity	833	1.00	1.76(1.11, 2.81)	1.77(1.10, 2.87)
Elevated blood pressure	678	1.00	2.90(1.76, 4.76)	3.07(1.86, 5.07)
Elevated triglycerides	406	1.00	1.44(0.87, 2.40)	1.49(0.89, 2.50)
Low HDL cholesterol	410	1.00	1.55(0.93, 2.58)	1.49(0.87, 2.55)
Elevated fasting glucose	651	1.00	1.70(1.06, 2.72)	1.73(1.08, 2.77)

† Model 1 adjusted for age.

‡ Model 2 adjusted for age, sex, high school education, urbanization, leisure-physical activity, alcohol consumption, and smoking.

Age and multivariate-adjusted odds ratios of MetS and its components according to 24 h UAE within normal range are shown in Table 3. Odds of MetS significantly increased across increasing quartiles of 24 h UAE within normal range (1.00, 1.13, 1.05 and 1.63, respectively; p for linear trend <0.001). Similarly, for components of MetS, the odds of central obesity, elevated blood pressure, and elevated triglycerides also significantly increased across increasing quartiles of 24 h UAE (1.00, 1.16, 1.24, and 1.73, respectively, for central obesity; 1.00, 1.35, 1.25 and 1.64, respectively, for elevated blood pressure; 1.00, 1.32, 1.08 and 1.51, respectively; p for linear trend <0.05 for all). The linear associations remained significant after multivariate adjustment for important risk factors (all p <0.05 for linear trends). However, no significant dose response relationship between 24 h UAE within normal range and elevated fasting glucose and low HDL cholesterol was found.

**Table 3:** Adjusted OR of metabolic syndrome and its components according to quartiles of 24 h UAE within normal range (Odds ratios and 95% confidence intervals).

	24 h UAE (mg)				p*
	Q1	Q2	Q3	Q4	
Metabolic syndrome					
Model 1†	1.00	1.13(0.82, 1.56)	1.05(0.76, 1.44)	1.63(1.20, 2.23)	<0.001
Model 2‡	1.00	1.11(0.80, 1.53)	1.02(0.74, 1.40)	1.58(1.16, 2.16)	0.002
Central obesity					
Model 1†	1.00	1.16(0.89, 1.52)	1.24(0.95, 1.61)	1.73(1.32, 2.25)	<0.001
Model 2‡	1.00	1.14(0.86, 1.50)	1.21(0.92, 1.59)	1.68(1.28, 2.22)	<0.001
Elevated blood pressure					
Model 1†	1.00	1.35(0.999, 1.82)	1.25(0.93, 1.67)	1.64(1.23, 2.20)	0.003
Model 2‡	1.00	1.35(1.001, 1.83)	1.26(0.93, 1.69)	1.68(1.25, 2.26)	0.002
Elevated triglycerides					
Model 1†	1.00	1.32(0.95, 1.84)	1.08(0.77, 1.50)	1.51(1.09, 2.08)	0.026
Model 2‡	1.00	1.32(0.95, 1.84)	1.06(0.76, 1.49)	1.47(1.06, 2.04)	0.04
Low HDL cholesterol					
Model 1†	1.00	0.95(0.69, 1.31)	0.85(0.62, 1.18)	1.03(0.75, 1.41)	0.72
Model 2‡	1.00	0.92(0.66, 1.28)	0.80(0.57, 1.12)	0.97(0.70, 1.34)	0.99
Elevated fasting glucose					
Model 1†	1.00	0.84(0.63, 1.12)	0.82(0.62, 1.08)	0.89(0.67, 1.17)	0.63
Model 2‡	1.00	0.83(0.62, 1.10)	0.80(0.61, 1.06)	0.88(0.66, 1.17)	0.61

*P values for linear trends.

† Model 1 adjusted for age.

‡ Model 2 adjusted for age, sex, high school education, urbanization, leisure-physical activity, alcohol consumption, and smoking.

Adjusted odds ratios of MetS and its components associated with 10 mg increase in 24 h UAE within normal range are presented in Table 4. Elevated 24 h UAE within normal range was significantly and positively associated with increased odds of MetS, central obesity, elevated blood pressure, and elevated triglycerides in both age- and multivariate-adjusted models. For each increment of 10 mg 24 h UAE within normal range, odds of MetS, central obesity, elevated blood pressure, and elevated triglycerides was 53%, 65%, 55%, and 41% higher, respectively.

**Table 4:** Adjusted OR of metabolic syndrome and its components associated with 10 mg increase in 24 h UAE within normal range (Odds ratios and 95% confidence intervals).

	Model 1†	Model 2‡
Metabolic syndrome	1.55(1.20, 2.00)	1.53(1.18, 1.98)
Central obesity	1.65(1.31, 2.09)	1.65(1.30, 2.10)
Elevated blood pressure	1.52(1.19, 1.95)	1.55(1.20, 1.99)
Elevated triglycerides	1.45(1.12, 1.89)	1.41(1.08, 1.83)
Low HDL cholesterol	1.06(0.81, 1.39)	1.04(0.78, 1.38)
Elevated fasting glucose	0.90(0.70, 1.15)	0.90(0.69, 1.16)

† Model 1 adjusted for age.

‡ Model 2 adjusted for age, sex, high school education, urbanization, leisure-physical activity, alcohol consumption, and smoking.

## Discussion

These data identify that not only microalbuminuria but also elevated 24 h UAE within the normal range was significantly associated with increased risk of MetS. Both prevalence of microalbuminuria and mean 24 h UAE significantly increased with number of metabolic risk factors. As expected, we found a significant dose response relationship between elevated 24 h UAE within normal range and risk of MetS, even after adjustment for multiple risk factors. It indicates that elevated 24 h UAE within normal range is also a stronger independent predictor of the risk of MetS. In addition, higher 24 h UAE was significantly associated with risk of components of MetS, including central obesity, elevated blood pressure, and elevated triglycerides. Our findings clearly illustrate the public health importance of the lowering of 24 h UAE for preventing MetS.

To our knowledge, this is the first study to report the association of elevated 24 h UAE within normal range with the risk of MetS in China. It has important clinical and public health implications because MetS is becoming common in the Chinese adults [1]. Accordingly, these findings contribute to the existing literature and provide new and important information in relation to the relationship between urinary microalbumin excretion and MetS in a representative sample of the Chinese general adult population and suggest that the lowering of 24 h UAE even within normal range should be an important priority for reducing the prevalence of MetS in China.

We noted that both the prevalence of microalbuminuria and the mean 24 h UAE significantly increased with number of metabolic risk factors, which is consistent with previous study[3, 5, 6, 8–10]. In addition, our study indicated that microalbuminuria was significantly associated with risk for MetS. To date, one study has attempted to quantify the association between microalbuminuria and risk of MetS in Chinese population, which reported that microalbuminuria was significantly associated with MetS (OR, 5.13; 95%CI, 1.96–13.45), consistent with our finding[5].

In present study we further examined the association between elevated 24 h UAE and risk of MetS by dividing participants into four groups according to quartiles of 24 h UAE within normal range. Our study indicated that the odds of MetS significantly increased with increase of 24 h UAE within normal range, which is in agreement with the findings of previous studies[3, 7–9]. To date, only one prospective cohort study has attempted to determine the association between urine albumin-to-creatinine ratio (UACR) estimated by a single morning urine sample and the development of MetS, which reported that compared with participants with first tertile of UACR, the hazard ratios (HRs) and 95% confidence intervals (CI) for MetS was 1.01 (0.71–1.45) and 1.57 (1.14–2.18) for participants in the second and third tertiles, respectively[7]. Moreover, we documented a strong dose-response association between 24 h UAE within normal range and risk of MetS in the general Chinese population. Note that this association is independent of age, sex, high school education, urbanization, leisure-physical activity, alcohol consumption, and smoking.

Our study found that microalbuminuria was significantly associated with high blood pressure and high blood glucose, in agreement with findings from previous studies[4, 5, 14]. In addition, our study documented a statistically significant relationship between microalbuminuria and central obesity, in line with previous study[5], but inconsistent with finding from Palaniappan and colleagues[4]. Interestingly, a statistically significant dose-response association was observed between elevated 24 h UAE within normal range and MetS components apart from elevated fasting glucose and low HDL cholesterol, which is in agreement with previous studies[3, 8].

Some potential limitations of our study should be noted. The cross sectional study design makes it hard to draw inferences regarding causal relationship between 24 h UAE and odds of MetS. In addition, the 24 h UAE estimated by a single 24-hour urine collection might have overestimated or underestimated the actual UAE in Chinese adult population. This random measurement error, owing to day-to-day variation in UAE in individuals, is possible to bias the association toward zero. Finally, lack of information on drug treatment for lipid abnormality (elevated triglycerides and reduced HDL-cholesterol) in the present study might result in a slight misclassification of the MetS. However, awareness, treatment and control rates of hypercholesterolaemia were extremely low in China. For example, the control rate was less than 2.0% in the Chinese population[15]. Nonetheless, the strengths of our study include its stringent quality control procedures; large sample size; using 24-hour urine collection considered as gold standard method for estimation of UAE; and careful measures of outcome variables.

In conclusion, our study identified both microalbuminuria and elevated 24 h UAE within normal range as stronger risk factors for MetS and also documented an independent and dose-response relation between 24 h UAE within normal range and odds of MetS. These findings suggest that the lowering of 24 h UAE should be a potential approach for reducing risk of MetS and its societal burden in China.
